# Do Stronger Employer Responsibilities Enhance Work Accommodation for Sick‐Listed Workers? Evidence From a Dutch Reform

**DOI:** 10.1002/hec.70038

**Published:** 2025-09-15

**Authors:** Laura Jansen, Viola Angelini, Max Groneck, Raun van Ooijen

**Affiliations:** ^1^ University of Groningen (RUG) Groningen the Netherlands; ^2^ University Medical Centre Groningen Groningen the Netherlands

**Keywords:** disability insurance, employer incentives, experience rating, workplace accommodation

## Abstract

This paper studies the impact of stronger employer responsibilities for facilitating work resumption of sick or disabled workers on employers' workplace accommodation efforts during sick leave. We exploit a reform in the Netherlands that altered experience rating—that is, shifting the costs of sick leave and disability insurance to the firm—both for permanent and non‐permanent employees. Using unique Dutch survey data on workplace accommodation of long‐term sick‐listed workers, we show that experience rating has no significant impact on accommodation efforts. Moreover, we provide evidence that the reform led to more firms opting for self‐arranging both the sick leave benefits and the reintegration process of sick non‐permanent workers, instead of using the public insurance scheme.

## 1 Introduction

Disability in the workforce negatively impacts employees' well‐being and career prospects while imposing a substantial financial burden on public finances through sick leave and disability benefits. In 2019, individuals with disabilities in the OECD faced a 27‐percentage‐point employment gap compared to their non‐disabled counterparts. Concurrently, public spending on sickness and disability benefits accounted for a significant 2% of GDP (OECD 2022, 2023). Employers can play a pivotal role in improving labor market outcomes for disabled workers, ultimately reducing public spending. They can do so by offering workplace accommodations such as task modifications, flexible scheduling, job coaching, training, or physical adjustments (e.g., ramps, accessible restrooms). Research shows that such accommodations facilitate a return to work, delay disability insurance (DI) applications, and support sustained employment (Burkhauser et al. 1995; Burkhauser et al. 1999; Campolieti 2005; Everhardt and de Jong 2011; Hill et al. 2016).

A key policy to encourage employers to provide accommodations is experience rating, which ties employers' insurance premiums to employees' sick leave and DI costs. While numerous studies have examined the effects of experience rating on labor market outcomes (De Groot and Koning 2016; Koning et al. 2025; Prinz and Ravesteijn 2020; Kyyrä and Paukkeri 2018; Kyyrä and Tuomala 2023; Van Sonsbeek and Gradus 2013), its direct influence on workplace accommodations—the mechanism it aims to activate—remains underexplored.

This paper investigates the impact of experience rating on workplace accommodations for long‐term sick‐listed workers. We leverage a 2013 Dutch reform, the Modernization Act for Sick Leave Benefits (BeZaVa), which introduced exogenous variation in experience rating. This reform extended experience rating to non‐permanent employees and tied its level to firm size, creating a natural experiment for a difference‐in‐differences (DID) analysis. Unique survey data from the National Social Insurance Institute (NSII, known as UWV in Dutch) targeted to 9‐month sick‐listed workers, allows us to examine workplace accommodations and employers' decisions to opt out of public sick leave insurance.

We focus on two key outcomes: the likelihood of workplace accommodations and firms' decisions to self‐manage sick leave benefits and reintegration processes for non‐permanent employees. Opting out of public insurance shifts the responsibility for disability benefit payments and reintegration from the NSII to the firm, although most firms choose to privately insure this risk after opting out. Firms that are well‐equipped to provide accommodations may find this approach more cost‐effective or efficient (Groenewoud et al. 2015).

We find three main results. We find no significant effect of experience rating on the likelihood of workplace accommodations for long‐term sick‐listed employees, regardless of their employment type, even though the financial incentives, especially for non‐permanent employees were substantial. One possible explanation is that employers perceive limited benefit from accommodation at such late stage compared to earlier interventions or preventative measures. Other reasons could include limited employer awareness of the reform or that the effect requires a longer time horizon to materialize. Since prior studies generally find that experience rating reduces DI inflow,^1^ it may also be that firms adopt other cost‐reducing strategies such as selective hiring of healthier workers or discouraging employees from calling in sick or applying to DI. One possibility we cannot rule out is that the effect is too small to detect with our sample size, even though all 9‐month sick‐listed workers in the Netherlands over a 2‐month period were invited to participate in the survey by the NSII. The 2015 wave, in particular, was fielded to monitor the BeZaVa reform.

Second, we find that experience rating significantly increases the likelihood of non‐permanent employees being employed by firms that opt out of public sick leave insurance. This suggests that firms may prefer self‐management or private insurance when public premiums are tied to reintegration outcomes. Opting out is positively associated with accommodation, either due to selection, that is, firms that already prioritize accommodate being more likely to opt out, or due to stronger financial incentives to reduce sickness costs that come with opting out itself, which may encourage firms to accommodate more. Finally, we observe that non‐permanent employees are less frequently accommodated than their permanent counterparts. For both groups, younger age, higher education, and employment at larger firms are positively correlated with workplace accommodations, while heart or vascular conditions are negatively associated.

This paper contributes to the literature in three key ways. First, it estimates the direct effect of experience rating on workplace accommodations and firms' sick leave insurance choices. Second, it expands on the limited research on experience rating and firms' insurance choices, which has only been examined in the studies by Groenewoud et al. (2015) and Hassink et al. (2018) for DI, while it has been researched more often for worker's compensation, see for example Morantz (2010). Understanding these choices is crucial, as they determine how reintegration is managed. Finally, we address a significant gap by including non‐permanent employees, a vulnerable group often excluded from such analyses despite their weaker labor market attachment and lower likelihood of receiving accommodations (Koning et al. 2025; Van der Burg 2011). Little evidence exists on the effectiveness of employer incentives for non‐permanent employees, even though they represent a large share of the workforce and are known to have substantially higher DI application risks than permanent employees (Koning et al. 2025). Unlike permanent employees, their contracts often end before the full sickness and reintegration trajectory is completed, reducing the potential payoff of firm investments in reintegration. Whether experience rating can improve employer behavior for this group of workers is therefore not self‐evident and an important policy question.

Our findings are relevant beyond the Netherlands and Finland, where experience rating is already implemented, as many countries face rising sick leave and disability challenges, especially with aging populations. In the United States, for instance, research suggests that nearly half of workers who would benefit from accommodations do not receive them, and about 20% of individuals in the disability system have remaining work capacity that could be utilized with proper accommodations (Maestas et al. 2014, 2019). Additionally, the US disability recipiency rate has risen over the past 4 decades (Burkhauser et al. 2016). Proposals to introduce employer incentives for addressing these challenges have been made on multiple occasions (D. Autor and Duggan 2010; D. H. Autor 2011; Burkhauser and Daly 2011; Liebman and Smalligan 2013). Thus, our results are relevant for countries exploring employer incentives to address workforce disability challenges. Moreover, while experience rating is limited in DI systems, it has a long history in unemployment insurance and worker's compensation programs (providing financial support and medical benefits to employees who are injured or become ill as a direct result of their job), particularly in the US, Canada, and Australia (see Koning (2016)). In these programs, the link between employer actions and total benefit claims is typically more direct (D. H. Autor 2011). For example, layoffs immediately affect unemployment claims, and workplace safety management directly influences the likelihood of injury‐related claims. By contrast, in DI systems, employers have more limited influence over whether employees develop work‐limiting health conditions in the first place. However, they can still influence DI entry by offering accommodations.^2^

The remainder of this paper is structured as follows: Section 2 reviews the literature on experience rating, while Section 3 outlines the Dutch institutional context and research design. Section 4 presents the data, and Section 5 introduces the empirical strategy. Section 6 details the results, which are discussed in Section 7 with robustness checks in Section 8. Finally, Section 9 concludes the paper.

## 2 Literature

Few studies theoretically analyze the employer's role in DI.^3^ A key assumption is employer moral hazard, where firms underinvest in retaining employees' work capacity due to public disability insurance. Experience rating aims to mitigate this by imposing costs on firms whose employees enter disability schemes, incentivizing accommodation efforts such as modifying tasks or equipment. However, as argued by Koning (2016), this can expose firms to significant financial risks, leading to selective hiring (as found by Hawkins and Simola (2020)), potential under‐reporting of disability, or even bankruptcies (De Groot and Koning 2022). Prinz and Ravesteijn (2020) and L. Jansen (2024) examine the optimal experience rating level, balancing these trade‐offs.

Empirical studies generally find that experience rating improves labor market outcomes, with no studies assessing its impact on accommodation. Studies on earlier Dutch reforms (Koning 2009; Van Sonsbeek and Gradus 2013) find that the introduction of experience rating reduces sick leave and DI inflow. De Groot and Koning (2016) find that the 2003 removal of experience rating for small firms increased DI inflow, but they find no effect from its reintroduction in 2008. They argue that this may be due to the earlier (2005) extension of the employer's obligation to cover sick pay for 2 years, which already introduced strong reintegration incentives. Koning et al. (2025), analyzing the BeZaVa reform, find that the reform as a whole had a large impact on DI inflow of non‐permanent employees, but estimate that the part of this effect attributable to experience rating is close to zero. Prinz and Ravesteijn (2020) also analyze the BeZaVa as a whole by comparing agency workers to permanent workers and find a large reduction in DI inflow. Finnish studies also find mainly positive effects of experience rating on labor market outcomes (Hawkins and Simola 2020; Kyyrä and Tuomala 2023; Korkeamäki and Kyyrä 2012), while Kyyrä and Paukkeri (2018)) finds no significant effect, although moderate‐sized effects could not be ruled out.

If accommodation is the main mechanism for these effects, it has to improve employment. Reviews (J. Jansen et al. (2021); Nevala et al. (2015)) generally find workplace accommodation reduces DI inflow and improves employment. Several studies have used survey data from the same source as the current study. Everhardt and de Jong (2011) found that vocational interventions by employers reduce the duration until reemployment of long‐term sick‐listed workers. More recently, Van Ooijen et al. (2024) showed that implemented disability‐related policies improve sustained employment among partially disabled workers. While these studies examine the effects of specific employer practices, our study focuses on whether financial incentives via experience rating influence firms' workplace accommodation behavior.

We also study the effect on opting out of public insurance. Groenewoud et al. (2015) find that 25% of employment agencies and 60% of other firms claims to have opted out of public sick leave insurance due to experience rating after the BeZaVa, anticipating lower costs through better reintegration. Hassink et al. (2018) show that Dutch firms with low DI risk are much more likely to opt out to private insurance, indicating firm self‐selection. Yet, no studies have quantitatively considered the impact of experience rating on this choice.

Although DI experience rating is unique to the Netherlands and Finland, similar employer incentives have been studied elsewhere. In Sweden, Hall et al. (2023) find more generous firm insurance increases sickness absence but does not affect selective hiring. In Austria, Böheim and Leoni (2020) find that abolishing compulsory sick leave insurance for blue‐collar workers reduces sickness absence without affecting hiring.

Finally, our study aligns with research on accommodation determinants. Hill et al. (2016) and Høgelund and Holm (2014) show that worker characteristics like age, education, tenure, and disability type influence accommodation provision. However, no research has examined whether these factors persist under strong employer incentives.

## 3 Institutional Context and Potential Effects of the Reform

### 3.1 Sick Leave and Disability Insurance in the Netherlands

In the Netherlands, employees who become sick—whether due to work‐related or non‐work‐related causes—are entitled to at least 70% of their gross wages for up to 2 years. After this period, they may apply for disability insurance (DI) benefits. Employers cannot dismiss workers or reduce wages during sick leave.^4^

The sick leave period differs for permanent and non‐permanent employees. For *permanent employees*, employers are responsible for both reintegration efforts and sick leave benefits. As public coverage is not available, they have the option to either self‐insure or purchase private insurance, transferring payment and reintegration responsibilities to insurers. For *non‐permanent employees*, the system differs. This group includes *agency workers*, whose employment terminates upon sickness, and *temporary workers*, whose contracts expire while on sick leave. Employers can use public sick leave insurance via the NSII, paying premiums while the NSII manages benefits and reintegration. Alternatively, firms can opt out of public insurance and take on direct responsibility both for payments and reintegration.

During the sick leave period, the legally required reintegration activities follow the Gatekeeper Protocol, which was introduced in 2002. After 6 weeks of sick leave, a problem assessment must be conducted. This involves an evaluation of the disability, how it affects the worker's tasks, which tasks can still be performed, and the goals of the upcoming reintegration activities. This assessment is conducted by the occupational health physician for permanent employees and by the NSII for non‐permanent employees. Two weeks later, an action plan based on this assessment must be created by both the employee and either the employer or the NSII. After 52 weeks, a first‐year assessment takes place. During this meeting, the employee and the employer or the NSII evaluate whether the reintegration is proceeding according to the action plan and whether new agreements need to be made.

After 2 years of sick leave, both permanent and non‐permanent employees may apply for DI benefits. The NSII's insurance physicians assess applicants' levels of disability and categorize them as eligible for partial DI benefits, full DI benefits, or ineligible. For partial DI, firms can opt out of public insurance for permanent employees but not for non‐permanent employees. We refer to the Dutch WGA scheme as partial DI for simplicity, although it includes both partial and permanent disability (35%–80% disability) and full but temporary disability (80%–100% disability). We refer to the Dutch IVA scheme as full DI (80%–100%) with no prospect of recovery, including only full and permanent DI. In most cases, firms choose to privately insure this risk rather than directly paying the benefits themselves (Koning 2016). All firms must insure full DI publicly.

### 3.2 The Reform and Its Expected Effects on Accommodation

The 2013–2014 Modernization Act of Sick Leave Benefits (BeZaVa) aimed to align financial incentives for permanent and non‐permanent employees (Tweede Kamer der Staten‐Generaal 2012). The reform had two key measures in 2014. A third measure affected non‐permanent employees exclusively and is not relevant to our difference‐in‐differences estimates. It involved stricter reintegration requirements and health criteria for extended sick leave. One of these measures is the introduction of the first‐year sick leave evaluation for non‐permanent employees, where the NSII assesses whether the employee still fulfills the requirements to be granted benefits (Tweede Kamer der Staten‐Generaal 2012).

The first measure is the extension of experience rating to public sick leave and partial DI premiums for *non‐permanent employees*, which were previously sector‐based and non‐individual. Experience rating incentivizes firms to internalize sick leave and partial DI costs, potentially encouraging workplace accommodations (Tweede Kamer der Staten‐Generaal 2012). These may range from additional breaks to workplace adjustments, task modifications, or job coaching (J. Jansen et al. 2023).

Experience‐rated premiums are based on the firm's sick leave or DI risk relative to the national average using data from 2 years prior. The DI risk is based on the total sick leave or DI benefit costs attributed to the firm 2 years prior, divided by the 5‐year average social insurance wage sum of the firm 2 years ago, compared to the average costs of all firms. The premium is calculated as follows, where the *calculation rate* and *correction rate* are set by the NSII (UWV 2014)^5^:

$${premium}_{i,t}={calculation\;rate}_{t}+{markup}_{i,t}$$

$${markup}_{i,t}={correction\;rate}_{t}\times \left({individual\;risk}_{i,t}-{average\;DI\;risk}_{t}\right)$$

$${individual\;risk}_{i,t}=\frac{{bene\mathrm{fi}t\;costs}_{i,t-2-s,}}{\sum_{s=0}^{5}{total\;insurance\;wage}_{i,t-2-s}/5}$$

These premiums are capped by a minimum and maximum premium and can reach up to 9% of total labor costs (De Groot and Koning 2016).

The second measure of the reform was to link the degree of experience rating to firm size. Before the reform, experience rating applied only to partial DI premiums for permanent employees, regardless of firm size. However, in practice, experience rating was more substantial for larger firms due to broader premium spread. The cutoff for being classified as a small firms was defined as 25 times the average social insurance wage. In 2013, the DI premium for small firms ranged from the minimum cap of 0.47% to the maximum cap of 1.56% of the wage sum, while for large firms the caps were 0.13% and 2.08% (UWV 2013). Hassink et al. (2015) show that between 2007 and 2011, small firms bore only 20% of the DI costs through increased premiums when a worker entered the DI scheme, due to the premium caps. In contrast, larger firms (with more than 25 workers) faced nearly the full cost, up to 100%. As a result, the removal of experience rating for permanent employees had a limited financial impact on small firms. Nevertheless, covering 20% of DI costs could represent a substantial financial burden for small firms. After the reform, these firms no longer had to bear this cost when a worker transitioned into DI.

Since the reform, experience rating has been based on firm size, defined by the annual social insurance wage bill relative to the national average. *Large firms* (wage bill > 100 times the national average) pay fully experience‐rated premiums based on their own risk. *Medium firms* (10–100 times the national average) pay a weighted average of the sector‐wide and of the individual experience‐rated premiums. *Small firms* (< 10 times the national average) pay only sector‐wide premiums.

Table 1 summarizes insurance types by employee category and firm size before and after the reform. The reform provides treatment variation to estimate the effects of introducing or removing experience rating (see Table 2). For non‐permanent employees, we compare those at large and medium firms (treatment group) to those at small firms (control group) to assess whether experience rating increases workplace accommodation. Firms internalizing sick leave and DI costs may reduce moral hazard and improve accommodations to shorten sick leave or prevent DI transitions.

**TABLE 1:** Insurance types by employee and firm size before and after reform.

Firm size/Type of contract	Small firm	Medium firm	Large firm
Permanent			
Before reform	Same rules for all firms:		
	1. No public sick leave insurance; employer pays benefits. Option to privately insure.		
	2. Public partial DI with experience‐rated premiums; option to opt out.		
	3. Obligatory full DI with non‐experience‐rated premiums.		
After reform	Partial DI premiums no longer experience‐rated.	Partial DI premiums partially experience‐rated.	*No changes.*
Non‐perm.			
Before reform	Same rules for all firms:		
	1. Public sick leave insurance available; option to opt out.		
	2. Obligatory public partial DI with non‐experience‐rated premiums.		
	3. Obligatory full DI.		
After reform	*No changes.*	Public sick leave and partial DI premiums partially experience‐rated.	Public sick leave and partial DI premiums fully experience‐rated.

**TABLE 2:** Treatment and control groups for the introduction and removal of experience rating.

	(Partial) introduction experience rating, sick leave, and partial DI premiums	(Partial) removal experience rating partial DI premiums
Treatment group	Non‐permanent employees at *medium* and *large* firms	Permanent employees at *small* and *medium* firms
Control group	Non‐permanent employees at *small* firms	Permanent employees at *large* firms

For permanent employees, we compare small and medium firms (treatment group) to large firms (control group) to examine the effects of removing experience rating. Since experience rating applied only to partial DI premiums for permanent employees, observed effects stem only from employer responses to anticipated financial consequences of DI transitions. Moreover, for permanent employees, the reform had a more modest financial impact, as the premium spread was already wider for larger firms pre‐reform (more than 25 times the average social insurance wage). By contrast for non‐permanent employees, the reform newly introduced experience rating and applied it to both sick leave and DI benefits, resulting in a much larger change in financial incentives.

### 3.3 The Reform's Expected Effects on Employer Sick Leave Insurance Choices

Experience rating may also influence firms' decisions to opt out of public sick leave insurance. Under the public scheme, firms pay experience‐rated premiums while reintegration is managed by the NSII, creating a potential misalignment between financial incentives and control over reintegration and costs. Opting out allows firms to avoid this misalignment. While some may resume reintegration responsibilities themselves, most contract private insurers to manage both reintegration and financial risks. These insurers often apply risk‐rated premiums, based on a large set of information elicited from the firms with the aim to align incentives more directly with reintegration outcomes. Premia for basic insurance vary greatly: the highest premium is four times higher than the lowest premium. In addition, private insurances can and do reject insurance applications (Aarts et al. 2018).

The decision to opt out is likely influenced by firms' risk characteristics. Firms with lower sick leave or DI risk, and those with more effective internal reintegration processes, may find private insurance cheaper and more efficient. In particular, low‐risk firms may view private insurance as more cost‐effective because it can offer full ex ante risk‐rating, unlike the public scheme that applies only partial experience rating due to minimum and maximum premium thresholds. Empirical evidence supports this: Groenewoud et al. (2015) found that 65% of employers who opted out after the BeZaVa reform did so with the expectation of improved reintegration outcomes, while Hassink et al. (2018) found that low‐risk firms were more likely to opt out and buy private insurance. Based on this, we hypothesize that experience rating increases opting out among medium and large firms, particularly for non‐permanent workers, due to the potential for lower costs and more efficient reintegration management by private insurers or the firm itself.

## 4 Data and Summary Statistics

### 4.1 Data

We use data from the Pathway to DI (Weg naar de WIA) survey, conducted in three waves (2008, 2012, 2015) administered by APE and Astri in collaboration with NSII. Each wave surveyed all eligible long‐term sick‐listed workers in the Netherlands over a 2‐month period. The final wave was specifically implemented to monitor the BeZaVa reform, which our study exploits. Each wave includes a follow‐up survey 18 months after sick leave began. The *full population* of 9‐month sick‐listed individuals was invited to participate, with a net response rate of about 35%. The survey, available on paper or online, includes permanent and non‐permanent employees as well as unemployed individuals. It provides detailed data on health, demographics, labor market outcomes, and employer accommodation.

Selection issues may arise as the survey occurs 9 months into sick leave, before any DI application, but after potential employer accommodations. If well‐accommodated workers return to work sooner, our sample could over‐represent firms less willing to accommodate. Additionally, voluntary participation may skew responses toward those with strong opinions on employer accommodations, though only 3.1% of cases are dropped for missing responses. However, since our identification strategy relies on a difference‐in‐differences design, any selection into survey participation should not bias the results as long as it does not vary systematically across survey waves between the treatment and control groups.

### 4.2 Sample Selection

We include only the main survey waves, excluding follow‐up responses, as early accommodations might reduce later sick leave duration. The sample consists of employed respondents aged 18–67, yielding an initial sample of 15,080 individuals, reduced to 12,523 after applying restrictions (Table 3). Details of the sample selection process are in Appendix A.

**TABLE 3:** Sample restrictions and sample size.

Sample	Nr. of respondents	% of initial sample
Initial sample of the population of interest in the three waves of the survey (employed individuals aged between 18 and 67)	15,080	100%
Sample restrictions		
1. Excluding respondents with missing key covariates.	13,679	90.7%
2. Excluding respondents who are in the “other” firm sector category.	13,673	90.7%
3. Excluding respondents who filled in the permanent survey as non‐permanent employee.	13,312	88.3%
4. Excluding respondents who answered that they became sick more than 1 month off from the inclusion period.	13,040	86.5%
5. Excluding respondents who did not answer the employer accommodation questions.	12,575	83.4%
6. Removing conflicting or missing values of firm size.	12,523	83.0%
Subsamples per employment type	Nr. of respondents	% of final sample
Non‐permanent employee.	3011	24.0%
Permanent employee.	9512	76.0%

*Note:* This table reports the sample restrictions and the accompanying sample sizes. It also reports the sample sizes of the two subsamples of non‐permanent and permanent employees.

### 4.3 Variables

The main dependent variables are employer accommodation, satisfaction with accommodation, and whether or not the firm opted out of public sick leave insurance for non‐permanent employees. Employer accommodation is a binary variable indicating whether any accommodation option was provided. The relevant survey question asks: *“What has your employer/employment agency done to get you either back to work or to retain you at work since you reported sick?”*. Response options include modified tasks, gradual return‐to‐work, workplace adjustments, other, or no accommodation. Since accommodations often involve multiple measures, we do not analyze them separately. Opting out is a binary variable that indicates whether a firm chose to opt out of public sick leave insurance for non‐permanent employees. This measure is not based on survey responses but derived from administrative records from NSII. Data on opting out are only available for the 2015 wave. Before 2015, all non‐permanent employees are coded as not opted out, while permanent employees are always missing in this variable.

Satisfaction with accommodation, used only in the descriptive analysis, measures whether respondents believe their employer did enough to facilitate their return. It is binary and considered only for accommodated individuals.

Independent variables include non‐permanent contract type (agency vs. temporary), demographics, disability type, firm characteristics, and year‐fixed effects. Demographics cover binary gender (male/female), education level (low, medium, high), migration background (binary), and age classes (18–35, 36–55, 56–60, 61–67). Education is categorized as low (primary/lower vocational), medium (secondary/senior vocational), or high (university/applied sciences).

Disability types include musculoskeletal, psychological, cardiovascular, and other conditions, with multiple responses allowed. A key firm characteristic considered is firm size, categorized as small (0–10 employees), medium (10–100 employees), and large (100+ employees). Since direct firm size data is unavailable for waves 2 and 3, this variable is derived from survey responses regarding the branch's size and whether it is part of a larger firm. Data from the first wave, where firm size information is available, confirms that 89% of such firms were large. Further details on the determination of firm size and robustness analyses using imputed measures are provided in Appendix A.

Firm sector (industry, transport, trade, services, public) is based on NSII records.

### 4.4 Descriptive Statistics

The upper panel of Table 4 presents summary statistics for non‐permanent (N = 3011), permanent (N = 9512), and total (N = 12,523) workers. Employer accommodation is more frequent among permanent employees (79%) than non‐permanent employees (29%). Additionally, non‐permanent employees are less satisfied with accommodations (58%) compared to permanent employees (81%), suggesting differences in both frequency and quality of accommodations.

**TABLE 4:** Summary statistics by type of employment contract.

	Non‐permanent workers	Permanent workers	Total
Sample size	3011 (24.0%)	9512 (76.0%)	12,523 (100.0%)
Workplace accommodation			
Workplace accommodation	0.303 (0.459)	0.793 (0.405)	0.675 (0.468)
Satisfaction with accommodation,	0.576 (0.494)	0.808 (0.394)	0.785 (0.411)
Conditional on being accommodated			
Type of non‐permanent contract			
Agency worker	0.214 (0.410)	—	—
Demographic variables			
Female	0.536 (0.499)	0.564 (0.496)	0.557 (0.497)
Migration background	0.227 (0.419)	0.122 (0.328)	0.148 (0.355)
Age class			
18–35	907 (30.1%)	1056 (11.1%)	1963 (15.7%)
36–55	1612 (53.5%)	5524 (58.1%)	7136 (57.0%)
56–60	356 (11.8%)	1990 (20.9%)	2346 (18.7%)
61–67	136 (4.5%)	942 (9.9%)	1078 (8.6%)
Education level			
Low education	1306 (43.4%)	3406 (35.8%)	4712 (37.6%)
Medium education	1179 (39.2%)	3086 (32.4%)	4265 (34.1%)
High education	526 (17.5%)	3020 (31.7%)	3546 (28.3%)
Disability type			
Musculoskeletal disability	0.507 (0.500)	0.376 (0.485)	0.408 (0.491)
Psychological disability	0.427 (0.495)	0.352 (0.478)	0.370 (0.483)
Heart/vascular disability	0.097 (0.296)	0.127 (0.333)	0.120 (0.325)
Other type of disability	0.307 (0.461)	0.375 (0.484)	0.359 (0.480)
Firm characteristics			
Opted out of public sick leave insurance	0.275 (0.447)	—	—
Firm size			
Small firm	332 (14.8%)	646 (6.8%)	978 (8.3%)
Medium firm	482 (21.6%)	1551 (16.3%)	2033 (17.3%)
Large firm	1422 (63.6%)	7315 (76.9%)	8737 (74.4%)
Firm sector			
Industry	395 (13.1%)	1528 (16.1%)	1923 (15.4%)
Transport	238 (7.9%)	526 (5.5%)	764 (6.1%)
Trade	412 (13.7%)	1116 (11.7%)	1528 (12.2%)
Services	1371 (45.5%)	1810 (19.0%)	3181 (25.4%)
Public	595 (19.8%)	4532 (47.6%)	5127 (40.9%)
Accommodation rates before and after reform			
Non‐permanent workers		Before reform	After reform
Small firms (control)		0.283 [0.217, 0.350]	0.250 [0.180, 0.320]
Medium/large firms (treatment)		0.268 [0.241, 0.294]	0.318 [0.287, 0.350]
Permanent workers			
Large firms (control)		0.699 [0.688, 0.710]	0.802 [0.786, 0.817]
Medium/small firms (treatment)		0.659 [0.647, 0.672]	0.767 [0.735, 0.799]

*Note:* The upper panel of this table presents summary statistics of our final sample of non‐permanent workers (column 1), permanent workers (column 2) and total sample (column 3). The mean and standard deviation (in brackets) are displayed for continuous variables, while the group frequencies and percentages (in brackets) are displayed for categorical variables. Data on opting out of public sick leave insurance is only provided for non‐permanent employees in wave 3 (2015). Also note that the disability types are not mutually exclusive, so the sum of the individual percentages does not add up to 100%. The bottom panel present the mean accommodation rates of non‐permanent and permanent employees, before and after the reform and by control and treatment group. The 95% confidence intervals of the means are shown in the brackets.

Women make up 56% of the sample, and most respondents (57%) are aged 36–55. Permanent employees are more often 55+ (31%) than non‐permanent employees (16%). Higher education levels are more common among permanent employees (32%) than non‐permanent employees (17%). The most prevalent disability type concerns musculoskeletal issues (41%), especially among non‐permanent employees (51%) compared to permanent employees (38%). Further descriptive statistics about firm characteristics shows that in 2015, 28% of non‐permanent employees worked at firms that opted out of public sick leave insurance. Permanent employees are more likely to work at large firms (77% vs. 64%), while non‐permanent employees are more common in the services sector (46% vs. 19%). Conversely, permanent employees are more often in the public sector (48% vs. 20%).

The bottom panel of Table 4 reports the average accommodation rates before and after the reform for both the treatment and control group. Among non‐permanent workers, for the treatment group (who were newly subject to experience rating) accommodation rates increased from 26.8% to 31.8%. In contrast, the control group (already exposed to experience rating) showed a decline. Yet, note that the confidence intervals of the mean appear wide which reflects the limited precision of these estimates. Among permanent workers, for the treatment group (for whom experience rating was removed or reduced), accommodation rates increased from 74.2% to 76.7%.while the control group saw a small decrease. These figures suggest that the introduction of experience rating for non‐permanent employees may have had a positive effect on accommodation, while the removal or reduction of experience rating for permanent employees had no clear effect.

## 5 Empirical Framework

Our empirical analysis consists of three parts. First, we provide correlational evidence on the determinants of workplace accommodation of long‐term sick‐listed non‐permanent and permanent employees using OLS regression. Next, we estimate the causal effects of the (partial) introduction and removal of experience rating on workplace accommodation and the chance of being employed at an employer who opted out of public sick leave insurance.

### 5.1 Determinants of Accommodation

To determine which types of workers are more likely to be accommodated and whether they are satisfied with it, we regress workplace accommodation and satisfaction with accommodation on our set of potential determinants. For the satisfaction regression, we only included respondents who indicated that they had been accommodated. We separate non‐permanent and permanent employees since their sick leave trajectories can be quite different, and therefore, we can expect different determinants.

### 5.2 Difference‐In‐Differences Analysis Workplace Accommodation

We use a DID strategy to estimate the effect of experience rating on workplace accommodation, focusing on the extensive margin. We employ two separate models for non‐permanent employees and permanent employees. To assess the impact of the (partial) introduction of experience rating, we compare non‐permanent employees at large and medium‐sized firms (treatment group) to non‐permanent employees at small firms (control group). For the removal of experience rating in partial DI premiums, we compare permanent employees at small and medium‐sized firms (treatment group) to permanent employees at large firms (control group). We assess the following linear probability models for our estimations of the effect of experience rating:

(1) $$\begin{array}{ll} {\text{Accom}}_{i,t} & =\beta_{0}+\beta_{1}\cdot {\text{After}}_{t}+\beta_{2}\cdot {\text{Large/medium}‐\text{sized}}_{i,t}\\ & +\beta_{3}\cdot {\text{After}}_{t}\cdot {\text{Large/medium}‐\text{sized}}_{i,t}+X_{i,t}^{\prime }\delta +\theta \cdot \text{Agency}+\varepsilon_{i,t} \end{array}$$

(2) $$\begin{array}{ll} {\text{Accom}}_{i,t} & =\beta_{0}+\beta_{1}\cdot {\text{After}}_{t}+\beta_{2}\cdot {\text{Small/medium}‐\text{sized}}_{i,t}\\ & +\beta_{3}\cdot {\text{After}}_{t}\cdot {\text{Small/medium}‐\text{sized}}_{i,t}+X_{i,t}^{\prime }\delta +\tau \cdot {\text{Wave}\;2}_{t}+\varepsilon_{i,t} \end{array}$$

with Equation (1) relating to non‐permanent employees and Equation (2) to permanent employees. The key coefficient of interest in both models, $\beta_{3}$, measures the causal effect of either the introduction or removal of experience rating. In these models, *Accom* is employer accommodation, *After* is a dummy that takes on the value of one if the observation took place after the reform (wave 3, 2015) and zero otherwise, *Large/medium‐sized* is a dummy that equals one if the respondent reports to have been employed at a large or medium‐sized firm and zero if the respondent is employed at a small firm, and the other way around for *Small/medium‐sized*. *X* is the vector of controls, consisting of our socio‐demographic variables (gender, migration background, age, and education level), disability type, and firm characteristics (firm size, firm sector). Next to these controls, the first equation includes *Agency*, a dummy that equals one if the non‐permanent employee is an agency worker and zero if the employee is a temporary worker. For Equation (2) only, we include the dummy for wave 2 (2012) as we have two pre‐treatment waves.

The parallel trend assumption can only be tested for permanent employees, not for non‐permanent employees. The reason is that firm size is only recorded in two pre‐reform waves (2008 and 2012) for permanent employees but only once for non‐permanent employees (in 2012). For permanent employees, we can assess the parallel trends assumption both graphically and analytically by estimating the DID model on the pre‐reform data. While we cannot empirically test this assumption for non‐permanent employees, there were no policy or contextual changes during the study period that would plausibly have affected non‐permanent employees differently based on firm size.

### 5.3 Difference‐In‐Differences Analysis Opting Out of Public Sick Leave Insurance

Next to potentially influencing accommodation, experience rating could also have had unintended effects on other outcomes, such as firms opting out of public sick leave insurance to circumvent the influence of NSII behavior on their now experience‐rated premiums. We test this by running a DID analysis on the likelihood of being employed at a firm that opted out, using non‐permanent employees at large and medium‐sized firms as treatment group and at small firms as control group. We estimate the following model:

(3) $$\begin{array}{ll} {\text{Opted out}}_{i,t} & =\beta_{0}+\beta_{1}\cdot {\text{After}}_{t}+\beta_{2}\cdot {\text{Large/medium}‐\text{sized}}_{i,t}\\ & +\beta_{3}\cdot {\text{After}}_{t}\cdot {\text{Large/medium}‐\text{sized}}_{i,t}+X_{i,t}^{\prime }\delta +\theta \cdot \text{Agency}+\varepsilon_{i,t} \end{array}$$

in which all the terms have the same meaning as Equations (1) and (2). In this analysis, as we have no data on opting out before the reform, we assume that no firms opted out before the reform. This assumption is reasonable as only 3% of all firms in the Netherlands had opted out in 2012 (Dumhs and Van Deursen 2017). Unfortunately, the lack of data on opting out before the reform also implies that we cannot assess the parallel trend assumption.

## 6 Results

### 6.1 Determinants of Accommodation Rates and Satisfaction

Table 5 presents the results of our analysis on the factors influencing the likelihood of receiving accommodation and satisfaction with accommodation. Columns 1 and 2 display OLS estimates for non‐permanent employees, focusing on the probability of receiving accommodation (column 1) and satisfaction with accommodation, conditional on receiving it (column 2). Columns 3 and 4 report similar results for permanent employees.

**TABLE 5:** Determinants of the rate of workplace accommodation and the satisfaction with it.

	Sample			
	Non‐perm.	Non‐perm.	Perm.	Perm.
	Dependent variable			
	Accom	Satisfaction	Accom	Satisfaction
Employment contract (base = temporary)				
Agency contract	−0.1173***	0.1245**		
	(0.0229)	(0.0557)		
Worker characteristics				
Female	0.0127	0.0405	0.0092	0.0263***
	(0.0182)	(0.0377)	(0.0090)	(0.0099)
Migration background	0.0015	−0.0650	−0.0180	−0.0249*
	(0.0199)	(0.0418)	(0.0130)	(0.0145)
Age class (base = 36–55)				
18–35	0.0165	0.0740*	0.0372***	−0.0171
	(0.0197)	(0.0378)	(0.0124)	(0.0151)
56–60	−0.0225	−0.0317	−0.0168	0.0121
	(0.0259)	(0.0575)	(0.0108)	(0.0116)
61–67	−0.0477	−0.0858	−0.0752***	0.0440***
	(0.0398)	(0.1002)	(0.0160)	(0.0160)
Education level (base = medium)				
Low	−0.0153	0.0576	−0.0504***	0.0021
	(0.0186)	(0.0406)	(0.0105)	(0.0111)
High	0.0794***	0.0960**	0.0173*	−0.0145
	(0.0254)	(0.0451)	(0.0100)	(0.0114)
Disability type				
Musculoskeletal	−0.0176	−0.0597	0.0018	−0.0544***
	(0.0192)	(0.0400)	(0.0107)	(0.0118)
Psychological	−0.0236	−0.1217***	−0.0068	−0.1064***
	(0.0188)	(0.0398)	(0.0105)	(0.0120)
Heart/vascular	−0.0562**	−0.1490**	−0.0281**	0.0290**
	(0.0281)	(0.0702)	(0.0141)	(0.0132)
Other	−0.0079	−0.0344	−0.0576***	0.0102
	(0.0199)	(0.0410)	(0.0103)	(0.0114)
Firm characteristics				
Opted out of public	0.1081***	−0.0796		
Sick leave insurance	(0.0341)	(0.0644)		
Firm sector (base = industry)				
Transport	−0.0585	0.0190	−0.0293	−0.0397*
	(0.0369)	(0.0788)	(0.0210)	(0.0227)
Trade	0.0128	−0.0358	−0.0261	−0.0338*
	(0.0338)	(0.0661)	(0.0166)	(0.0173)
Services	−0.0307	−0.0025	−0.0348**	−0.0077
	(0.0288)	(0.0610)	(0.0146)	(0.0150)
Public	0.0437	−0.0084	0.0015	−0.0262*
	(0.0329)	(0.0644)	(0.0126)	(0.0134)
Firm size (base = medium)				
Small			−0.0357*	0.0000
			(0.0207)	(0.0203)
Large			0.0358***	−0.0166
			(0.0118)	(0.0121)
Wave dummies (base = wave 1)				
Wave 2 (2012)	−0.0928***	−0.2750***	0.0049	−0.0986***
	(0.0211)	(0.0406)	(0.0102)	(0.0111)
Wave 3 (2015)	−0.0986***	−0.2594***	0.0066	−0.0928***
	(0.0244)	(0.0478)	(0.0101)	(0.0109)
Constant	0.4093***	0.7936***	0.8176***	0.9422***
	(0.0377)	(0.0729)	(0.0188)	(0.0194)
Observations	3011	821	9512	7443

*Note:* This table presents OLS estimates for the likelihood of employer accommodation and, conditional on accommodation, binary employee satisfaction. Firm size is excluded as a regressor for non‐permanent employees due to its absence in wave 1, to avoid reducing the sample size. Heteroskedasticity‐robust standard errors are in parentheses. Significance levels: *** p<0.01, ** p<0.05, * p<0.1.

The findings reveal notable differences in accommodation rates between non‐permanent and permanent employees, with non‐permanent employees being accommodated less frequently. However, the determinants of accommodation are broadly similar for both groups. For non‐permanent employees, those with agency contracts are less likely to receive accommodation than those with temporary contracts. Gender has no significant effect on accommodation likelihood for either group, but among permanent employees, women report greater satisfaction with accommodation than men when it is provided. Migration background does not significantly impact the likelihood of accommodation for either group, although satisfaction appears somewhat lower among employees with a migration background.

An age‐related pattern emerges, with younger workers more likely to receive accommodation than older workers. This may suggest that employers perceive a lower return on accommodating workers closer to retirement. Conversely, older permanent employees report lower satisfaction with accommodation. Educational attainment also plays a significant role: individuals with lower levels of education are less likely to be accommodated and are less satisfied when accommodation is provided. These results align with Hill et al. (2016), who found that higher education positively influences employer accommodation.

Disability characteristics further influence accommodation and satisfaction outcomes, with similar patterns for both contract types. Workers with heart and vascular conditions are least likely to receive accommodation and are least satisfied when accommodations are made. This may be due to the inherent difficulty of accommodating such conditions. Employees with psychological issues also report lower satisfaction with accommodations.

Firm characteristics and timing additionally play a role. While the sector of the firm has no significant effect, smaller firms are less likely to accommodate their employees. Non‐permanent workers employed by firms that opted out of public sick leave insurance are more likely to receive accommodation. Interestingly, reported accommodation rates were higher in 2008 compared to 2012 or 2015, despite the financial crisis in 2008, which might have been expected to negatively impact accommodation practices.

In conclusion, older, less‐educated workers, and those with specific disabilities are less likely to receive accommodations, a pattern seen in both permanent and non‐permanent employees. While not causal, these findings highlight significant disparities in accommodation practices, with some groups receiving more favorable treatment.

### 6.2 Effect of Experience Rating on Workplace Accommodation

#### 6.2.1 Parallel Trend Accommodation

**FIGURE 1:**
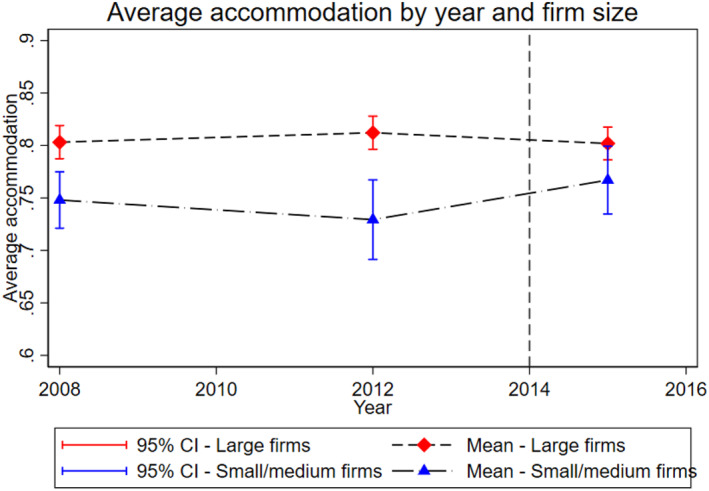
Workplace accommodation rate trends of permanent employees. This figure displays the average accommodation rate and its 95% confidence interval of permanent employees at large versus small and medium‐sized firms in the three waves (2008, 2012 and 2015).

To inspect the parallel trend assumption of our DID analysis, we plot pre‐treatment trends and perform a placebo test on our sample of permanent employees at large and small firms. Figure 1 shows the average accommodation levels of the treated and untreated permanent workers from 2008 to 2015. The average accommodation rates do not diverge significantly, and treated workers (at small and medium‐sized firms) even show an upward trend in accommodation. Table 6 reports the placebo test estimates. The estimate of the placebo treatment indicator (Wave 2*Small or medium firm) is statistically insignificant. Hence, the parallel trend assumption appears to hold for permanent employees. As noted earlier, we cannot test the parallel trend assumption for non‐permanent employees, but we have no reason to believe it differs from them, as we are not aware of any major policies or other factors that could have affected the relative difference between non‐permanent workers at large versus small and medium‐sized firms.

**TABLE 6:** Placebo test DID model permanent employees.

	Dependent variable	
	Accom	Accom
	Sample	
	Perm.	Perm.
Wave 2 (2012)	0.0090	0.0101
	(0.0114)	(0.0114)
Small/medium‐sized	−0.0551***	−0.0433***
	(0.0159)	(0.0161)
Wave 2 * small/medium‐sized	−0.0277	−0.0273
	(0.0262)	(0.0259)
Wave 3 (2015)	−0.0012	0.0036
	(0.0113)	(0.0115)
Wave 3 * small/medium‐sized	0.0202	0.0129
	(0.0242)	(0.0243)
Constant	0.8031***	0.8533***
	(0.0080)	(0.0170)
Controls	No	Yes
2008 included	Yes	Yes
Observations	9512	9512

*Note:* This table reports the DID results of Equation (2) for permanent employees, but includes the placebo‐treatment coefficient Wave 2*Small firm. The controls in column 2 are the same as column 3 of Table 5, excluding firm size as a separate control as this is already in the treatment indicator, and wave 3 is included as After, while wave 2 is included as separate wave dummy. Heteroskedasticity‐robust standard errors are reported in parentheses. Significance levels: *** p<0.01, ** p<0.05, * p<0.1.

#### 6.2.2 Difference‐In‐Differences Results

Now, we examine the effect of experience rating on workplace accommodation. The difference‐in‐difference regressions confirm what we see in Table 4, yet find no significant effects. Columns 1 and 2 of Table 7 present the results for the introduction of experience rating for non‐permanent employees Equation (1). Since the inclusion of controls does not significantly alter the coefficients, we focus on the model with controls. The estimated effect of introducing experience rating is 6.24% points, a notable figure given the average accommodation rate for non‐permanent employees is around 30%. However, this effect is not statistically significant. The imprecision arises from the large standard errors associated with our limited sample size. Although we do not find a statistically significant effect, we cannot rule out the possibility of a limited effect due to the statistical power of our analysis.

**TABLE 7:** Main DID results of the introduction and removal of experience rating.

	Dependent variable			
	Accom	Accom	Accom	Accom
	Sample			
	Non‐perm.	Non‐perm.	Perm.	Perm.
After	−0.0333	−0.0257	−0.0056	0.0006
	(0.0486)	(0.0486)	(0.0098)	(0.0112)
Large/medium‐sized	−0.0155	−0.0155		
	(0.0362)	(0.0359)		
After * large/medium‐sized	0.0837	0.0624		
	(0.0530)	(0.0528)		
Small/medium‐sized			−0.0660***	−0.0536***
			(0.0125)	(0.0128)
After * small/medium‐sized			0.0311	0.0232
			(0.0222)	(0.0222)
Constant	0.2833***	0.3594***	0.8075***	0.8559***
	(0.0336)	(0.0478)	(0.0057)	(0.0169)
Controls	No	Yes	No	Yes
2008 included	No	No	Yes	Yes
Observations	2236	2236	9512	9512

*Note:* The first two columns show DID results on the *introduction* of experience rating Equation (1) for long‐term sick‐listed *non‐permanent* employees at small and large firms in waves 2 and 3 (wave 1 is excluded due to missing firm size data). The last two columns present DID results on the *removal* of experience rating Equation (2) for *permanent* employees at small and large firms in waves 1–3. The dependent variable is a binary indicator of employer accommodation. For columns 1 and 2, controls match those in column 1 of Table 5, excluding opting out and separate wave dummies (except for the 2015 dummy “After”). For columns 3 and 4, controls follow column 3 of Table 5, omitting firm size (since it is in the treatment indicator) and including wave 3 as “After” with wave 2 as a separate dummy. Heteroskedasticity‐robust standard errors are in parentheses. Significance levels: *** p<0.01, ** p<0.05, * p<0.1.

Columns 3 and 4 of Table 7 report the results of removing experience‐rated partial DI premiums for permanent employees at small firms (Equation 2). Once again, the inclusion of controls (column 4) does not materially affect the coefficients in column 3, so we focus on the controlled model. This analysis estimates a 2.32% point *increase* in the likelihood of accommodation when employers are no longer required to pay experience‐rated partial DI premiums. However, this effect is also statistically insignificant, though the standard errors are somewhat smaller due to the larger sample size. Since the removal of experience rating has smaller financial consequences for firms employing permanent workers than the introduction of experience rating had for those employing non‐permanent workers, this result is less surprising. Small and medium‐sized firms already faced less complete experience rating for their permanent employees before the reform, due to stricter caps on the premiums. Additionally, the introduction of experience rating for non‐permanent employees applied not only to partial DI but also to sick leave premiums.

Overall, we do not find significant evidence that either the introduction or removal of experience rating has a meaningful impact on workplace accommodation for long‐term sick‐listed workers. However, these findings should not be interpreted as evidence that experience rating is always ineffective at encouraging employers to increase accommodation efforts. Our study focuses on workers who have been sick for at least 9 months, a group for which employers may not perceive accommodation as likely to reduce sick leave duration or DI inflows. For other categories of workers—such as those at risk of becoming sick‐listed or those newly sick‐listed—experience rating might be more effective. This aligns with previous research showing a negative relationship between experience rating and sick leave or DI inflows (De Groot and Koning 2016; Hawkins and Simola 2020; Korkeamäki and Kyyrä 2012; Kyyrä and Paukkeri 2018; Prinz and Ravesteijn 2020; Van Sonsbeek and Gradus 2013), suggesting that experience rating can indeed enhance accommodation efforts. In Section 7, we explore possible alternative explanations for why accommodation rates do not increase, even though prior research generally finds that experience rating reduces DI inflow and increases employment.

Finally, in Supporting Information S1: Appendix A1 we explore the effects of the reform on specific types of accommodation. Generally, we find insignificant results on the grouped categories of accommodation. We do find evidence of substitution from workplace or equipment adjustments toward adjustments in work hours following the removal of experience rating for permanent employees.

### 6.3 Effect of Experience Rating on Public Sick Leave Insurance Choice

Table 8, column 1, reports the estimated treatment effect of introducing experience rating on the likelihood of having an employer who opted out of public sick leave insurance. We only observe whether a firm opted out of public sick leave insurance for non‐permanent employees, but not out of public partial DI or for permanent employees. The estimated effect is 28% points, statistically significant at the 1% level. This is a substantial effect, especially considering that only 3% of firms had opted out in 2012 (Dumhs and Van Deursen 2017).

**TABLE 8:** DID results on opting out of public sick leave insurance.

	Dependent variable	
	Opted out	Opted out
	Sample	
	Non‐perm.	Non‐perm.
After	0.0724***	0.0790***
	(0.0210)	(0.0206)
Large/medium‐sized	0.0000	−0.0109*
	(0.0000)	(0.0061)
After * large/medium‐sized	0.2398***	0.2352***
	(0.0265)	(0.0258)
Constant	−0.0000	−0.0081
	(0.0000)	(0.0213)
Controls	No	Yes
2008 included	No	No
Observations	2236	2236

*Note:* The table reports the estimated coefficients of Equation (3), that is, the DID analysis of the introduction of experience rating on being at an employer who opted out of public sick leave insurance. The control group consists of non‐permanent employees at small firms and treatment of non‐permanent employees at large and medium‐sized firms. The controls are the same as column 1 of Table 5, except for the exclusion of opting out as a control and the exclusion of separate wave dummies (except for After, the wave dummy for 2015). We assume that no firms had opted out before the reform (2012). Heteroskedasticity‐robust standard errors are reported in parentheses. Significance levels: *** p<0.01, ** p<0.05, * p<0.1.

One potential concern is that the survey data are collected at the employee level, while the decision to opt out is made at the firm level and applies to all employees. However, if the focus is on the number of employees working for an employer that opted out, rather than the number of firms opting out, the analysis remains appropriate. This is further supported by the fact that the survey targeted the entire population of 9‐month sick‐listed workers. Moreover, data from the NSII indicate that 31% of the total wage sum in 2015 was associated with employers who had opted out, compared to only 7% in 2013. These aggregate figures align with our estimate, further reinforcing the validity of the results.

The findings suggest that experience rating creates a strong incentive for employers to opt out of public insurance. This could reflect dissatisfaction with the reintegration services provided by the NSII, or a belief among firms that either they or private insurers can manage reintegration more effective and cost‐efficient.

Relating this finding to accommodation, we observe that opting out and the likelihood of accommodation are positively associated. The regression coefficient for opting out is 10.8% (see Table 5), suggesting that being employed at a firm that has opted out is associated with a 10.81% points higher likelihood of being accommodated. However, this association should be interpreted with caution, as we cannot disentangle whether the relationship reflects a causal effect of opting out or selection effects, that is, whether firms that already prioritize accommodation are more likely to opt out.

While we do not classify the increase in firms opting out as inherently positive or negative, it could have significant implications for the public insurance system. If low‐risk firms predominantly opt out, high‐risk firms remain in the public sick leave system, potentially increasing its financial strain. Conversely, if firms that opt out provide better reintegration and accommodation than those that remain, this could lead to improved workplace environments for employees.

## 7 Discussion: Alternative Explanations Unchanged Accommodation Rates

Although most literature finds an effect of experience rating on DI inflow or employment, we do not find a significant effect of experience rating on the likelihood of accommodation for long‐term sick‐listed employees. Here, we explore different reasons as to why this may be the case.

A first explanation could be that experience rating is ineffective for long‐term sick‐listed workers because employers view accommodation measures as less effective at this stage than in the early phases of sickness or during prevention. By 9 months of sick leave, many of the initial reintegration or accommodation activities, such as the legally required problem assessment and the action plan, may already have taken place. Employers of non‐permanent employees may also be more concerned with the newly introduced first year sick leave evaluation, which occurs at 12 months, rather than on introducing further or adjusted accommodation at this late phase of sick leave.

Another possibility is limited employer awareness of the financial consequences of long‐term sick leave or DI inflow. For instance, Koning (2009) found that a rise in DI premiums due to experience rating led to a 15% reduction in DI inflow the following year, suggesting that employers may not have been well informed either about the system of experience rating or the firm's disability risk exposure. Although this study was based on data from 2000–2002, shortly after the initial introduction of experience rating in the Netherlands in 1998, more recent findings suggest that awareness remained limited years later. Groenewoud et al. (2015) reported that in 2014–2015, 21% of employment agencies and 50% of other firms were not or minimally familiar with the experience rating system for non‐permanent employees. The limited awareness may have weakened the potential effect of the reform, particularly for non‐permanent employees. A counterargument can, however, be made here as Prinz and Ravesteijn (2020) and Koning et al. (2025) find that the BeZaVa reform as a whole had a large and immediate impact on DI inflow. This would imply that employers were clearly aware of the reform and adjusted accordingly to reduce DI inflow. Yet, these papers focus on the reform as a whole, including the stricter reintegration and monitoring requirements for non‐permanent employees. Therefore, it could be that the awareness or understanding of the other measures exceeded that of experience rating, and that the responses to these measures resulted in lower DI inflow. Koning et al. (2025) also show evidence that most of their observed effect is driven by the monitoring measures rather than experience rating.

Related to employer awareness, it is also possible that the effect of the reform emerges only in the long term. This may be because employers were initially unaware of the reform and became informed over time, or because they were aware from early on but needed time to adapt their accommodation policies and practices. Yet again, the same counterargument applies, as the reform was found to have an immediate impact on DI inflow. However, it is possible that the reintegration and monitoring measures produced immediate effects, while the effects of experience rating through accommodation may take longer to become visible.

Yet, since the general literature finds a reduction in the DI inflow as response to experience rating, if there is no effect on accommodation, this could suggest that firms find other behaviors to reduce costs. Such behaviors could be selective hiring of healthier workers or putting more pressure on workers not to call in sick or apply to DI. As argued by Koning (2016), experience rating could lead to underreporting of sickness as a result of pressure by the employer. For worker's compensation, it has also recently been found that employer responsibility and experience rating can lead to claim suppression in Canada (Premji et al. 2025).

Finally, it remains possible that a small effect exists that falls below the detection threshold given our sample size, even though all 9‐month sick‐listed workers were invited to participate by the NSII. Given the careful study design to monitor the BeZaVa reform, issues of limited statistical power were not expected.

## 8 Robustness Checks

We check the robustness of our DID results in a variety of ways. A first concern might be that in our analyses, we pool two types of non‐permanent workers together: agency workers and workers with an ending temporary contract. It could be that this decision drives our results as these two types might have a different relation to their employer. This would mean that pooling them into one specification is not appropriate. To address this concern, we split the main DID analysis on non‐permanent employees by type of contract. We do this for both dependent variables (accommodation and opted out). Table A2 in Appendix B reports all robustness checks on the DID analysis on workplace accommodation, while Appendix Table A3 covers those on opting out of public sick leave insurance. In Appendix Table A2, the first column reports the DID estimate on accommodation using the subsample of workers with an ending contract and the second column reports those of workers with an agency contract. This does not affect our results—our DID estimate remains insignificant. In Appendix Table A3, we do the same but now with opting out of public sick leave insurance as dependent variable. Column 1 reports the DID results on the sample of workers with an ending temporary contract and column 2 on the sample of agency workers. The DID coefficient remains statistically significant at the 1% level. The effect size remains relatively stable, as it is 21.75% points for temporary workers and 25.6% points for agency workers, compared to the pooled DID estimate of 23.52% points. Hence, we do not see a substantial difference between temporary and agency workers.

Another potential limitation could arise from people who switch employers or become self‐employed during the sick leave period, as this can cause confusion about the firm accommodation question and change the employer's incentives. In Table A2 of Appendix B, column 3 (non‐permanent workers) and column 4 (permanent workers) report the DID estimate on accommodation without these employees. The DID coefficient remains statistically insignificant. In Appendix Table A3, column 3, we also take these employees out of the sample for the analysis on opting out of public sick leave insurance. Our DID estimate virtually remains the same.

During the 9 months of sick leave, some employees may have received a different contract type. This might distort the accuracy of our results since this changes the employer incentives. Specifically, this concerns permanent employees who stayed at their initial employer during the 9 months but received a non‐permanent contract, and non‐permanent employees who changed to a permanent contract. The first group is not recorded in our data, but the second group is. To safeguard against this distortion, we exclude these individuals from our analysis. We do this for our DID on accommodation for non‐permanent employees in Appendix Table A2, column 5 and for opting out in Appendix Table A3 column 4. This exercise does not have any sizable affect our previously found results.

Finally, to verify that our results are not driven by our model specification in terms of treatment group selection, we use alternative specifications based on Koning et al. (2025) and Prinz and Ravesteijn (2020). Koning et al. (2025) estimate the effect of experience rating on DI inflow comparing non‐permanent employees at small firms to large firms. When we apply this treatment group definition ‐ excluding the medium firms and comparing large and small firms ‐ our DID analyses on accommodation and opting out show again no significant effect of introducing experience rating on accommodation (see Appendix Table A2, column 6, and Appendix Table A3, column 5. For accommodation, we find a larger point estimate (9.25% points) that is significant but only at the 10% level. For opting out, we also find an even larger point estimate of 30.2% points. This seems to show that the effect is even larger for firms who are exposed to full rather than partial experience rating.

Koning et al. (2025) also examine the effect of the total reform (so the alignment of financial incentives and the monitoring measures) by comparing all non‐permanent employees to all permanent employees in a DID analysis. Column 7, Appendix Table A2 shows that the point estimate of the effect of the total reform on the accommodation rate of non‐permanent workers compared to permanent workers is even slightly negative, but remains insignificant.^6^

Prinz and Ravesteijn (2020) compare agency workers at large firms to all permanent workers to estimate the effect of experience rating on DI inflow, also exploiting the BeZaVa reform. In column 8, Appendix Table A2 the estimates of a DID analysis imitating their treatment and control groups are reported.^7^ We find a significant (at the 1% level) and very sizable positive effect: a 15.14% points increase in the average accommodation rates of agency workers at large firms, compared to permanent workers. However, this result should be interpreted with caution. First, the sample of agency workers at large firms is very small, especially compared to the control group. Second, the control group is not a clean control as the incentives for employers of permanent employees were also altered during this period—experience rating was partially eliminated for small and medium‐sized firms.

To summarize, our results remain robust against a variety of robustness checks. On one occasion, our results are not the same. Replicating the model specifications of Prinz and Ravesteijn (2020) does change our results, but we argue that this specification is not suited to our research question.

## 9 Conclusion

The employment rate of disabled individuals in OECD countries remains significantly lower than that of individuals without disabilities, while public expenditure on sickness and disability accounts for a substantial portion of GDP (OECD 2022, 2023). Employers play a crucial role in addressing these challenges by creating disability‐inclusive workplaces to enhance the labor market outcomes of disabled workers. This paper examines the impact of extending a government‐mandated employer incentive—experience rating—to non‐permanent employees on workplace accommodation efforts for long‐term sick‐listed employees.

Our analysis reveals no statistically significant effect of shifting the costs of sick leave and partial DI toward or away from firms through experience rating on employer accommodation for 9‐month sick‐listed workers, regardless of whether they hold permanent or non‐permanent contracts. However, we find a significant increase in the number of employers of non‐permanent employees (specifically, those at large and medium‐sized firms) opting out of public sick leave insurance after the reform. Additionally, our results highlight notable patterns in the determinants of accommodation. While non‐permanent employees are less frequently accommodated, the factors influencing accommodation for both non‐permanent and permanent employees are largely similar.

These findings carry important implications for countries grappling with firm moral hazard—namely, the reluctance of firms to support sick‐listed and partially disabled workers. Our results suggest that experience rating does not achieve the desired effect of increasing accommodation rates for long‐term sick‐listed workers. This raises questions about whether employer responsibility should extend to such workers or perhaps only to the initial stages of sickness or disability. This holds particularly given that the severity of their disabilities may limit the effectiveness of accommodation efforts and that in response, employers may seek other measures to reduce DI inflow such as selective hiring or pressuring workers not to apply. Further research is needed to determine whether experience rating improves preventive accommodation efforts or reintegration measures for short‐term sick‐listed workers. Moreover, further research could examine how BeZaVa affected DI inflow and employment for long‐term sick‐listed workers changed as result of experience rating. This would help clarify whether employers responded to the reform through alternative channels other than accommodation. Additionally, the limited effectiveness of experience rating may stem from a lack of awareness among firms, highlighting the potential benefit of initiatives aimed at increasing employer understanding of the system or simplifying the system. Simplifying the system is also one of the major recommendations of a recent policy report covering the entire Dutch DI system (OCTAS 2024).

Moreover, our findings indicate that firms opting out of public sick leave insurance are more likely to provide accommodations, reflecting possible dissatisfaction with the public reintegration process. This suggests that a system in which firms bear financial responsibility through experience rating while reintegration is managed by public entities may not be optimal. Transferring both financial responsibility and reintegration efforts to firms could merit consideration.

Finally, policymakers should carefully consider our findings on the determinants of accommodation, which suggest disparities in the likelihood of employees receiving accommodations. Ensuring equitable access to accommodation should remain a priority in designing policies to address these issues, for instance through providing accommodation subsidies or more support for firms that deal with workers with sickness or disability.

## Conflicts of Interest

The authors declare no conflicts of interest.

## Supporting information


Supporting Information S1

## Data Availability

The data that support the findings of this study are openly available in DANS Data Station Social Sciences and Humanities at https://doi.org/10.17026/dans‐zeu‐576n.

## Endnotes

^1^
The NSII also used the same survey as we do and found that for long‐term sick‐listed non‐permanent employees, the employment rate of agency workers 1.5 years after the start of sick leave increased between 2012 and 2015, while no change was observed other non‐permanent workers (Rijnsburger 2017). Although this study does not employ a causal identification strategy, it provides suggestive evidence that experience rating may have improved employment outcomes.

^2^
For a review of experience rating in Worker's Compensation, see for example Tompa et al. (2012), Tompa et al. (2013); and for experience rating in Unemployment Insurance, see for example Anderson and Meyer (2000), Auray and Fuller (2020), and Mongrain and Roberts (2005).

^3^
Most of the literature focuses on workers' moral hazard, where key policy parameters are screening strictness and the generosity of sick leave and DI benefits. See for example, Godard et al. (2024) or Ahammer and Packham (2025) on screening and Böckerman et al. (2018) and Mullen and Staubli (2016) on replacement rates.

^4^
If an employee on a temporary contract reaches the end of their contract during sick leave, the National Social Insurance Institute (NSII) assumes responsibility for benefits.

^5^
The social insurance wage is the portion of wages subject to social insurance contributions.

^6^
We cannot run this robustness check for opting out as it involves permanent employees, who do not have public sick leave insurance.

^7^
Again, we cannot run this robustness check for opting out as it involves permanent employees.

## Appendix A Sample Selection and Cleaning

**TABLE A1:** Main DID results using multiple imputation on firm size.

	Dependent variable		
	Accom	Accom	Opted out
	Sample		
	Non‐perm.	Perm.	Non‐perm.
After	−0.0085	0.0051	0.1390***
	(0.0421)	(0.0115)	(0.0242)
Large/medium‐sized	0.0255		−0.0094*
	(0.0308)		(0.0056)
After * large/medium‐sized	0.0470		0.1798***
	(0.03004)		(0.0300)
Small/medium‐sized		−0.0526***	
		(0.0124)	
After * small/medium‐sized		0.0163	
		(0.0212)	
Constant	0.3271***	0.8550***	−0.0134
	(0.0447)	(0.0169)	(0.0220)
Controls	Yes	Yes	Yes
2008 included	No	Yes	No
Observations	2236	9518	2236

*Note:* This table reports the main DID analyses using multiple logit imputation on firm size. The first column estimates the effect of the introduction of experience rating on accommodation of non‐permanent employees, column 2 the effect of the removal of experience rating on accommodation of permanent employees, while column 3 considers the effect of experience rating on opting out of public sick leave insurance for non‐permanent workers. The number of imputed datasets is 10 for all three analyses. Imputation‐corrected and heteroskedasticity‐robust standard errors are reported in parentheses. Significance levels: *** p<0.01, ** p<0.05, * p<0.1.

Our first sample restriction limits the sample to those respondents who filled in the key covariates. These consist of, firstly, the specific groups: non‐permanent employees (distinguishing two subcategories, agency workers and temporary workers) and permanent employees. Moreover, gender, education level, age, type of disability, and migration background must be filled in. Firm size must also be reported. Yet, firm size was not asked about in the non‐permanent survey of wave 1. Therefore, we allow these respondents to have a missing firm size variable.

The second restriction excludes respondents whose firm sector is categorized as “other”, as these are only six observations.

The third sample restriction is to exclude those respondents who reported to be an agency worker or temporary worker but filled in the permanent worker survey. Fifth, we exclude the respondents who entered that their first date of reported sickness was more than one month away from the inclusion period (for wave 2, October 2011 and November 2011, and for wave 3, October 2014 up to and including January 2015^8^).

Next, we restrict the sample to those who have answered the question which informs our key dependent (binary) variable of whether accommodation was provided by the employer.

Finally, we clean and impute values for firm size. For observations in the second and third wave, the size of the total firm is missing. Therefore, there we assume that the total firm is large (more than 100 employees) when an employee works at a branch that is part of a larger firm. Moreover, when it was not indicated whether the branch was part of a large firm, but all other questions in that part of the survey were filled in, we code this as a “no” for being part of a larger firm. When this question is missing but size of the total firm is filled in, we code this as a “yes” on whether the branch is part of a larger firm. Then, still, 14 values for whether the branch is part of a large firm are still missing are still missing on. These are deleted. Moreover, 12 observations are conflicting and therefore dropped: they claim not to be part of a larger firm but total firm size is filled in. Finally, we drop 6 conflicting observations where total firm size is smaller than the branch size, and 20 observations from wave 1 where total firm size was asked for but not answered when a respondent indicated that they were part of a larger firm.

We test whether our results depend on the choice to assume that everyone who indicated that the branch was part of a larger firm was employed at a large firm. We do so by multiple imputation on a logit estimation on both Small∗Medium−sized or Large∗Medium−sized using our controls and complete information on total firm size from wave 1, permanent employees. For this estimation, we do not drop the conflicting observations where total firm size is smaller than the branch size but rather as this makes the branch size a perfect predictor for total firm size when we split the two groups into small versus medium or large. We take 10 imputed datasets. Table A1 reports the DID estimates on accommodation for non‐permanent employees (column 1), permanent employees (column 2), and for opting out for non‐permanent employees (column 3). This does not substantially differ from our main results without multiple imputation. The effect of experience rating on accommodation remains significant while the effect on opting out reduces from 23.5% to 18.0%, but remains significant at the 1% level.

## Appendix B Robustness Checks Tables

**TABLE A2:** Robustness checks accommodation.

	Dependent variable							
	Accom	Accom	Accom	Accom	Accom	Accom	Accom	Accom
	Robustness check							
	Temporary workers	Agency workers	No change in contract	No change in contract	No switch to perm. Contract	No medium‐sized firm	Replicating KMP	Replicating PR
After	−0.0268	−0.0058	−0.0099	−0.0044	−0.0278	−0.0242	0.0036	0.0045
	(0.0526)	(0.1311)	(0.0511)	(0.0113)	(0.0488)	(0.0484)	(0.0088)	(0.0089)
Large/medium‐sized	0.0068	−0.1296	0.0126		−0.0172			
	(0.0398)	(0.0842)	(0.0377)		(0.0359)			
After * large/medium	0.0468	0.1301	0.0375		0.0655			
	(0.0576)	(0.1386)	(0.0557)		(0.0530)			
Small/medium‐sized				−0.0484***				
				(0.0130)				
After*Small/medium				0.0263				
				(0.0224)				
Large						−0.0418		
						(0.0368)		
After * large						0.0925*		
						(0.0541)		
Non‐perm.							−0.4759***	
							(0.0121)	
After * non‐perm.							−0.0038	
							(0.0197)	
TreatmentPR								−0.6485***
								(0.0266)
After * TreatmentPR								0.1514***
								(0.0529)
Constant	0.3518***	0.3401	0.3401***	0.8561***	0.3530***	0.3651***	0.8311***	0.8400***
	(0.0518)	(0.2309)	(0.0505)	(0.0170)	(0.0479)	(0.0512)	(0.0145)	(0.0157)
Controls	Yes	Yes	Yes	Yes	Yes	Yes	Yes	Yes
2008 included	No	No	No	Yes	No	No	No	No
Observations	1820	416	1948	9234	2220	1754	12,523	9781

*Note:* The table presents our main DID analyses on accommodation using different samples and treatment group definitions. Columns 1 and 2 examine non‐permanent employees, distinguishing between temporary (1) and agency workers (2). Columns 3 and 4 exclude those who change employers or become self‐employed, for non‐permanent (3) and permanent employees (4). Column 5 excludes non‐permanent employees who switch to permanent contracts. Columns 6 and 7 follow Koning et al. (2025), comparing large versus small firms (6) and estimating the full reform effect by comparing permanent to non‐permanent workers (7). Column 8 follows Prinz and Ravesteijn (2020), comparing agency to permanent employees. Heteroskedasticity‐robust standard errors are in parentheses. Significance levels: *** p<0.01, ** p<0.05, * p<0.1.

**TABLE A3:** Robustness checks opting out.

	Dependent variable				
	Opted out	Opted out	Opted out	Opted out	Opted out
	Robustness check				
	Temporary worker	Agency worker	No switch in contract	No switch to perm. Contract	No medium‐ sized firm
After	0.0287*	0.3513***	0.0808***	0.0804***	0.0797***
	(0.0152)	(0.1020)	(0.0226)	(0.0210)	(0.0206)
Large/medium‐sized	−0.0080*	0.0060	−0.0099	−0.0108*	
	(0.0044)	(0.0187)	(0.0063)	(0.0061)	
After * large/medium‐sized	0.2175***	0.2560**	0.2378***	0.2322***	
	(0.0223)	(0.1118)	(0.0282)	(0.0260)	
Large					−0.0148**
					(0.0073)
After * large					0.3020***
					(0.0276)
Constant	0.0094	−0.0649	−0.0088	−0.0064	0.0112
	(0.0214)	(0.1870)	(0.0229)	(0.0215)	(0.0257)
Controls	Yes	Yes	Yes	Yes	Yes
2008 included	No	No	No	No	No
Observations	1820	416	1948	2220	1754

*Note:* The table presents our main DID analyses on opting out using different samples and treatment group definitions. Columns 1 and 2 focus on non‐permanent employees, distinguishing between temporary (1) and agency workers (2). Column 3 excludes those who change employers or become self‐employed during sick leave, while Column 4 excludes those who switch to permanent contracts. Column 5 follows Koning et al. (2025), comparing non‐permanent employees in large and small firms.
