# Supplementary material for: Do Stronger Employer Responsibilities Enhance Work Accommodation for Sick‐Listed Workers? Evidence From a Dutch Reform
Source: Health Econ. 2025 Sep 15;34(12):2297–316. doi: 10.1002/hec.70038 (PMC12579522; doi:10.1002/hec.70038)
Supplement: Supplementary file 1 — Supporting Information S1 [file HEC-34-2297-s001.pdf]

Online Appendix

September 8, 2025

Additional DID clustered accommodation types

To explore whether the effects of the reform differed by specific type of accommodation, we estimate DID models using five grouped accommodation categories as dependent variables. We follow the classification of Hill et al. (2016), who distinguish between three categories: task or job adjustments, hours adjustments, and adjustments to the workplace or equipment. We add a fourth category for work on a therapeutic basis/gradual return to work and a fifth 'other' category, which includes, among others, job-oriented education/training and conflict resolution or mediation. We conduct DID analyses on each accommodation type separately, for both non-permanent employees (Table 1) and permanent employees (Table 2).

For non-permanent employees, Table 1 shows a significant (at the 5% level) increase of nearly 8 percentage points in the chance of receiving the 'other' type of accommodation. For permanent employees, we find that the removal of experience rating was associated with an increase in work hours adjustments of approximately 5 percentage points and a decrease in adjustments to the workplace or equipment of approximately 3 percentage points. This may reflect a shift from more costly to less costly forms of accommodation. Accommodations are often provided in combination: 43% of accommodated workers received more than one type. To examine the intensive margin of accommodation, we also estimate DID models using the number of accommodation types received as the dependent variable (Table 3). These analyses show no statistically significant effects, in line with the main analysis.

Table 1: *DID results non-permanent employees types of accommodation*

	Dependent variable				
	diff./fewer tasks	fewer hours	therap. work	workpl./equipm.	other
	Sample				
	Non-perm.	Non-perm.	Non-perm.	Non-perm.	Non-perm.
After	-0.0072	-0.0377	0.0064	0.0105	-0.0195
	(0.0281)	(0.0278)	(0.0306)	(0.0182)	(0.0331)
Large/medium	0.0171	-0.0136	0.0308	0.0017	-0.0458*
	(0.0214)	(0.0231)	(0.0223)	(0.0119)	(0.0248)
After * Large/medium	-0.0075	0.0221	0.0016	-0.0162	0.0774**
	(0.0309)	(0.0306)	(0.0341)	(0.0194)	(0.0357)
Constant	0.0953***	0.0735**	0.1606***	0.0031	0.1344***
	(0.0296)	(0.0287)	(0.0311)	(0.0149)	(0.0321)
Controls	Yes	Yes	Yes	Yes	Yes
2008 included	No	No	No	No	No
Observations	2,236	2,236	2,236	2,236	2,236

Notes: This table shows the DID results for non-permanent employees on the different possible types of accommodations, which are binary variables taking the value of 1 if that accommodation was offered and zero if it was not, including the possibility of no other accommodation being provided or a different type of accommodation.

Table 2: *DID results permanent employees types of accommodation*

	Dependent variable				
	diff./fewer tasks	fewer hours	therap. work	workpl./equipm.	other
	Sample				
	Perm.	Perm.	Perm.	Perm.	Perm.
After	0.0832***	0.0307**	0.0379***	0.0181**	-0.0578***
	(0.0129)	(0.0125)	(0.0140)	(0.0083)	(0.0095)
Small/medium	-0.0442***	-0.0466***	-0.0336**	-0.0106	0.0097
	(0.0130)	(0.0125)	(0.0149)	(0.0084)	(0.0109)
After * Small/medium	0.0279	0.0534**	-0.0041	-0.0296**	-0.0191
	(0.0240)	(0.0233)	(0.0264)	(0.0142)	(0.0169)
Constant	0.3047***	0.2698***	0.5772***	0.1036***	0.1107***
	(0.0189)	(0.0183)	(0.0208)	(0.0122)	(0.0144)
Controls	Yes	Yes	Yes	Yes	Yes
2008 included	Yes	Yes	Yes	Yes	Yes
Observations	9,512	9,512	9,512	9,512	9,512

Notes: This table shows the DID results for permanent employees on the different possible types of accommodations, which are binary variables taking the value of 1 if that accommodation was offered and zero if it was not, including the possibility of no other accommodation being provided or a different type of accommodation.

Table 3: *DID results intensive margin of accommodation*

	Dependent variable	
	Number of accom. types	Number of accom. types
	Sample	
	Non-perm.	Perm.
After	-0.0537	0.1487***
	(0.1397)	(0.0266)
Large/medium	0.0075	
	(0.1081)	
After * Large/medium	0.0041	
	(0.1531)	
Small/medium		-0.0508*
		(0.0273)
After * Small/medium		-0.0062
		(0.0496)
Constant	1.3083***	1.5860***
	(0.1389)	(0.0394)
Controls	Yes	Yes
2008 included	No	Yes
Observations	641	7,539

Notes: This table reports the DID results on the intensive margin of accommodation, i.e. the number of different accommodation types summed up, conditional on being accommodated. Column 1 reports this for non-permanent employees and column 2 reports this for permanent employees.

References

Hill, M. J., Maestas, N., and Mullen, K. J. (2016). Employer accommodation and labor supply of disabled workers. *Labour Economics*, 41:291–303.
